# Diversity matters — extending sound intensity coding by inner hair cells via heterogeneous synapses

**DOI:** 10.15252/embj.2023114587

**Published:** 2023-10-06

**Authors:** Tobias Moser, Nare Karagulyan, Jakob Neef, Lina María Jaime Tobón

**Affiliations:** ^1^ Institute for Auditory Neuroscience and InnerEarLab University Medical Center Göttingen Göttingen Germany; ^2^ Auditory Neuroscience and Synaptic Nanophysiology Group Max Planck Institute for Multidisciplinary Sciences Göttingen Germany; ^3^ Cluster of Excellence “Multiscale Bioimaging of Excitable Cells” Göttingen Germany; ^4^ Hertha Sponer College Cluster of Excellence “Multiscale Bioimaging of Excitable Cells” Cluster of Excellence Göttingen Germany

**Keywords:** auditory system, cochlea, hair cell, spiral ganglion neuron, synapse, Neuroscience

## Abstract

Our sense of hearing enables the processing of stimuli that differ in sound pressure by more than six orders of magnitude. How to process a wide range of stimulus intensities with temporal precision is an enigmatic phenomenon of the auditory system. Downstream of dynamic range compression by active cochlear micromechanics, the inner hair cells (IHCs) cover the full intensity range of sound input. Yet, the firing rate in each of their postsynaptic spiral ganglion neurons (SGNs) encodes only a fraction of it. As a population, spiral ganglion neurons with their respective individual coding fractions cover the entire audible range. How such “dynamic range fractionation” arises is a topic of current research and the focus of this review. Here, we discuss mechanisms for generating the diverse functional properties of SGNs and formulate testable hypotheses. We postulate that an interplay of synaptic heterogeneity, molecularly distinct subtypes of SGNs, and efferent modulation serves the neural decomposition of sound information and thus contributes to a population code for sound intensity.

## Graphical abstract

This review discusses mechanisms and proposes novel hypotheses for how the mammalian hearing organ can encode an exceptionally wide range of sound intensities and how sound intensity information is decoded.

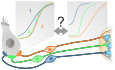

## Introduction

**Figure 1:**
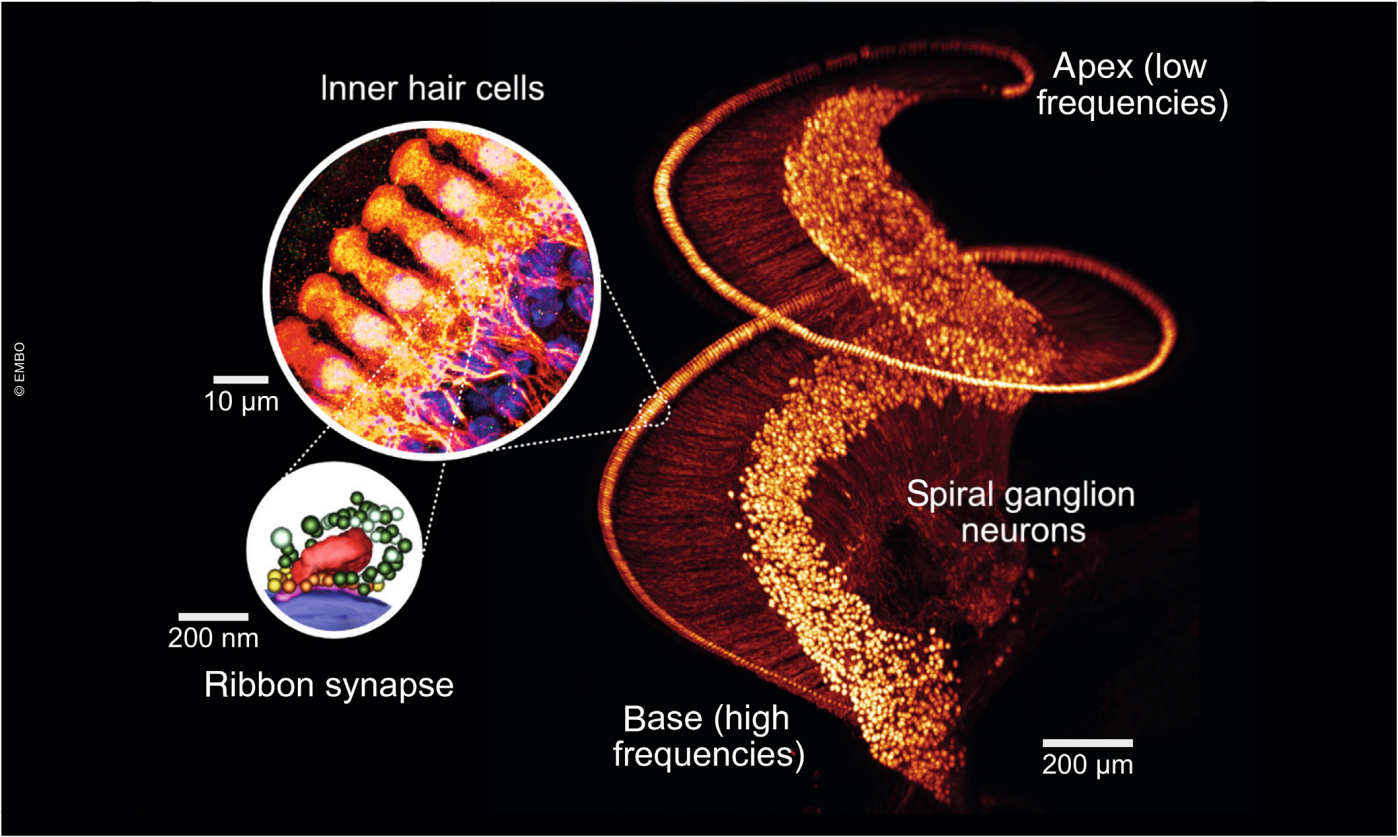
Afferent cochlear circuitry This composite graphic displays a mouse cochlea rendered transparent, immunofluorescently labeled for the IHC and SGN context marker parvalbumin, and imaged by light sheet microscopy (taken from cover of Michanski *et al*, 2019). The large inset shows the IHC‐SGN contacts from a confocal stack imaged at higher magnification, and the smaller inset represents a 3D reconstruction of an IHC active zone based on electron tomography, with the synaptic ribbon in red, ribbon‐proximal synaptic vesicles in green and membrane‐proximal vesicles in yellow and gold (from Chakrabarti *et al*, 2022).

**Figure 2:**
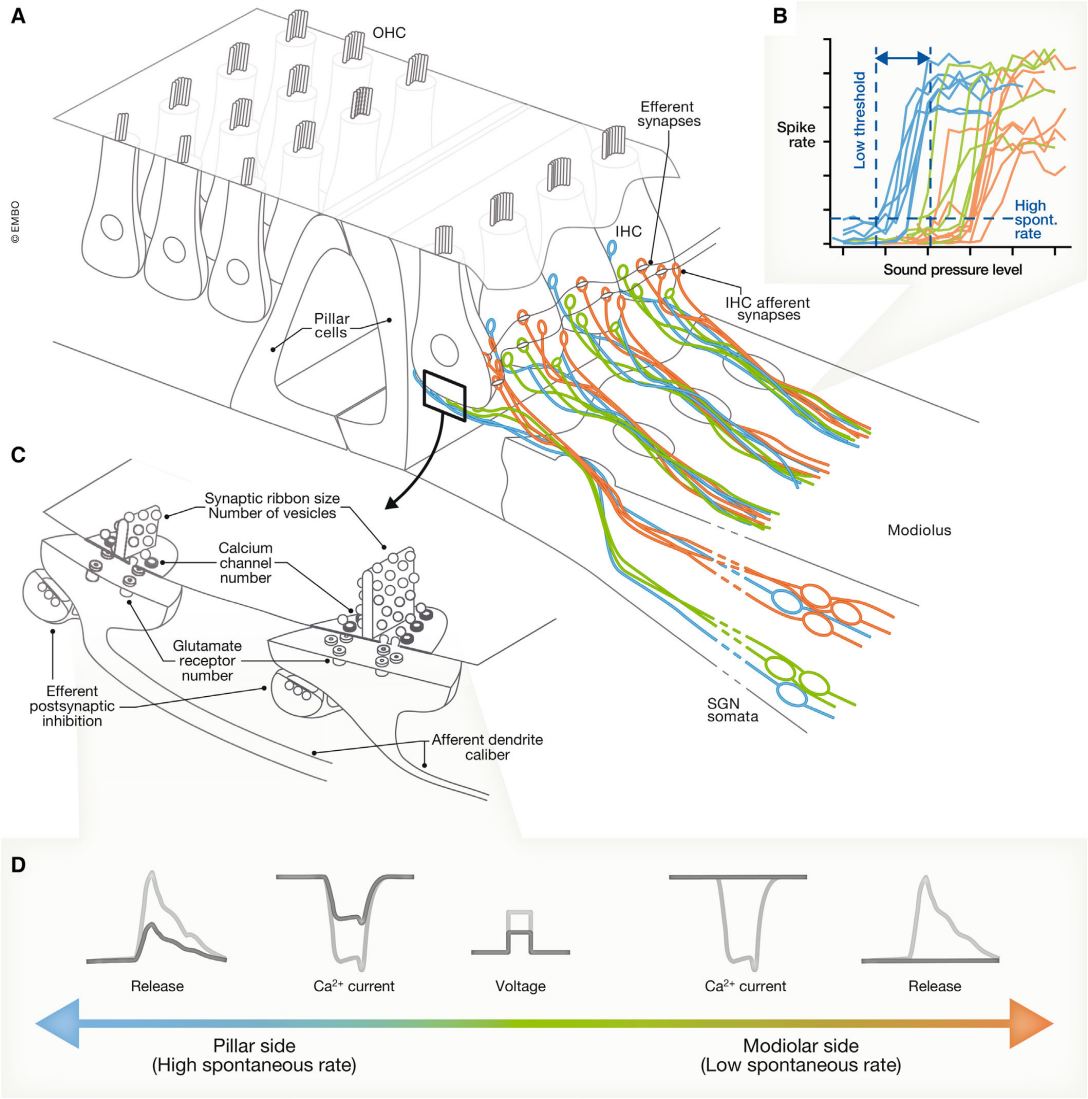
Varying properties of inner hair cell active zones (A, B) Organ of Corti with three rows of outer hair cells (OHCs) and one row of IHCs (A). The latter are innervated by peripheral neurites of type I SGNs with different spontaneous and sound‐evoked firing properties (B, modified from Ohn *et al*, 2016, putative assignment to molecular subtypes Ia–c) with their synapses distributed along the pillar–modiolar IHC axis according to their properties as reported by Charles Liberman. They likely correspond to the molecularly distinct type Ia–c SGN subtypes. (C) Inset to A with a schematic of afferent IHC‐SGN synapses and corresponding efferent synapses onto the afferent postsynaptic SGN. Active zones differ even within the same IHC: larger presynaptic active zones with greater Ca_V_1.3 channel clusters tend to be at the modiolar side. (D) Active zones differ also functionally: e.g., pillar ones activate at more negative potentials and, moreover, show tighter Ca^2+^ nanodomain coupling of Ca_V_1.3 channels to vesicular release sites than the average modiolar ones. (A) and (C) modified from Meyer & Moser (2010).

Our sense of hearing is critical for our vocal communication. Impaired speech comprehension requires intervention in approximately 466 million people with disabling hearing loss worldwide (WHO, 2019). Our ability to hear acoustic signals as different as rustling leaves and a roaring jet engine, i.e., sound pressures (intensities) that differ by about six orders of magnitude, and to process them with exceptional temporal precision, arguably, is one of the most fascinating yet enigmatic phenomena of the auditory system. Sound intensity coding at the level of single SGNs faces the so‐called dynamic range problem (Evans, 1981): While the receptor potential of IHCs covers the full intensity range of sound input (Russell & Sellick, 1978; Russell, 1983; Cheatham & Dallos, 2000), SGNs change their spike rate only over a fraction of the input range (Kiang *et al*, 1965; Sachs & Abbas, 1974; Liberman, 1978; Winter *et al*, 1990; Taberner & Liberman, 2005; Huet *et al*, 2016). In inner hair cells (IHCs), the ensuing mechanoelectrical transduction generates a graded receptor potential (stronger depolarization from stronger sound intensity) activating voltage‐gated Ca^2+^ influx that triggers the release of synaptic vesicles. This results in efficient, temporally precise, and indefatigable transmission at the IHCs' 5–30 specialized ribbon‐type synapses with type I spiral ganglion neurons (SGNs, Figs 1 and 2C; Meyer & Moser, 2010; Fettiplace, 2017; Moser *et al*, 2019; Rutherford *et al*, 2021). Sound of different frequencies activates IHCs, and consequently postsynaptic SGNs, at different locations along the length of the cochlea. This way, information about sound frequency is primarily represented as a place code, i.e., a tonotopic mapping via the identity of the activated SGNs. As the traveling wave gets wider with stronger sound intensities, a larger set of SGNs will be activated around the tonotopic position resulting in a population code for intensity. At the level of single SGNs at any tonotopic position of the cochlea, sound intensity is represented in spike rate‐ and time‐codes. Matching the large input dynamic range to limited output range of the SGNs involves a number of fascinating biological mechanisms: (i) Outer hair cell electromotility that amplifies weak basilar membrane vibration and dampens strong vibrations; (ii) adaptation processes of hair cells, synapses, and neurons; and (iii) tiling of the dynamic range by spiral ganglion neurons (SGNs) with diverse intensity coding.

**Figure 3:**
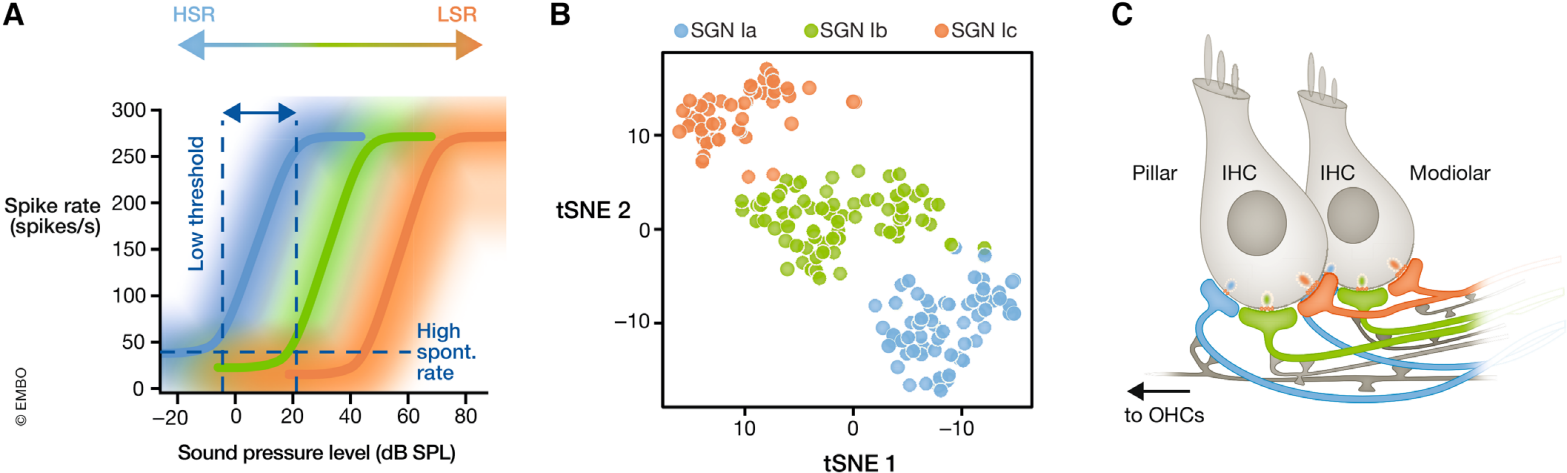
Neural candidate mechanisms of cochlear wide range sound intensity encoding (A) SGNs with diverse discharge rate‐sound pressure level functions fractionate the dynamic range (modified from Ohn *et al* (2016), putative assignment to molecular subtypes Ia–c). SGNs with high spontaneous rate (HSR) and low sound threshold, suggested to correspond to type Ia SGNs, preferentially innervate the pillar IHC side, while SGNs with low spontaneous rate (LSR) and high threshold preferentially innervate the modiolar side. This has been postulated to result from operation of the corresponding IHC active zones at more negative potentials. (B) SGNs differ in their molecular profiles and can be clustered into three subtypes (Ia–c), thought to correspond to high (Ia), intermediate (Ib), and low SR (Ic) functional phenotypes (modified from Shrestha *et al*, 2018). tSNE: t‐distributed stochastic neighbor embedding, a technique for dimensionality reduction. (C) SGN function is differentially modulated by efferent synapses of lateral (LOC) and medial (MOC) olivocochlear fibers, which originate from the lateral or medial part of the superior olivary complex, respectively. MOC fibers also innervate outer hair cells (OHCs) (modified from Hua *et al*, 2021).

Specifically, SGNs that share the same frequency tuning and might therefore receive input from the same IHC differ in the range of sound pressures over which they change their firing rate (Figs 2A and B and 3A). Yet, as a population, SGNs cover the entire audible range with their individual coded fractions. How such “dynamic range fractionation” arises is a topic of current research and will be at the focus of this review.

**Figure 4:**
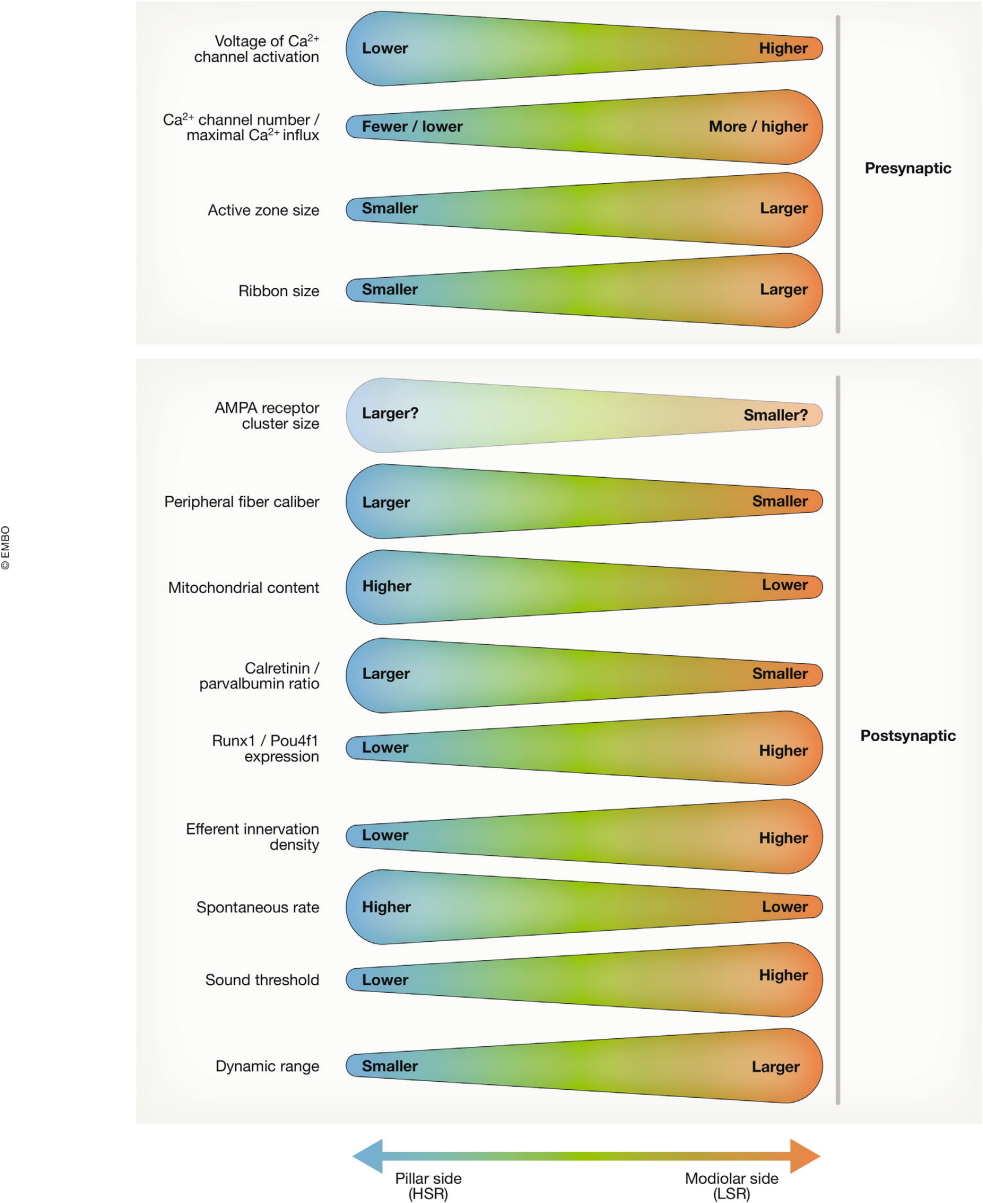
SGNs and their afferent synapses differ according to the position of the afferent synapse on the IHC Properties of afferent synapses (upper section) as well as of SGN morphology and function (lower section) exhibit gradients along the pillar–modiolar axis of the inner hair cell.

A first indication of how the signal from IHCs can lead to intensity‐dependent activation of different SGNs was provided through seminal analysis by Liberman *et al*, who showed a correlation between the sensitivity of a SGN to sound and its contact point on the IHC: SGNs with high spontaneous firing rate (SR) and low sound threshold (“high SR” SGNs) tend to innervate the “pillar” side of the IHC (facing the pillar cells toward the outside of the cochlear spiral), while low SR, high‐threshold (“low SR”) SGNs preferentially synapse on the opposite “modiolar” side (facing the cochlear modiolus on the inside of the cochlear spiral; Fig 2A and B; Liberman, 1982; Merchan‐Perez & Liberman, 1996). How such diverse SGN coding properties and spatial segregation on the IHC membrane are achieved remains to be elucidated. Three major hypotheses have been put forward (Guinan, 2018; Moser *et al*, 2019; Shrestha & Goodrich, 2019): (i) IHCs decompose the intensity information into complementary neural codes by varying the properties of their presynaptic active zones (AZs, Figs 2C and D and 4; Frank *et al*, 2009; Meyer *et al*, 2009; Grant *et al*, 2010; Kantardzhieva *et al*, 2013; Ohn *et al*, 2016; Michanski *et al*, 2019; Hua *et al*, 2021; Niwa *et al*, 2021; Özçete & Moser, 2021), (ii) different molecular SGN profiles shape diverse SGN firing properties (Fig 3B; Davis & Crozier, 2016; Petitpré *et al*, 2018; Shrestha *et al*, 2018; Sun *et al*, 2018; Li *et al*, 2020; Markowitz & Kalluri, 2020; Siebald *et al*, 2023), (iii) efferent innervation differentially modulates afferent synapses (Fig 3C; Ruel *et al*, 2001; Yin *et al*, 2014; Wu *et al*, 2020; Hua *et al*, 2021).

Additionally, glial (or supporting) cells of the cochlea might contribute to shape synaptic or firing properties of SGNs as is observed for regulation of synaptic strength in the brain (Letellier *et al*, 2016). We hypothesize that an interplay of the aforementioned mechanisms results in fractionated coding of the audible intensity range by individual SGNs in order to achieve good sound intensity discrimination also for low sound intensities (Ehret, 1975). We review evidence for these candidate mechanisms and potential relationships between them. In addition, we propose experiments to evaluate their causal contributions to sound intensity coding. Advances in the experimental and theoretical analysis of molecular profiles, anatomy, and physiology of SGNs and IHCs (Moser *et al*, 2019; Shrestha & Goodrich, 2019), as well as technological advances such as multiscale imaging and optogenetic stimulation of the cochlea, now enable combinatorial approaches to this fascinating phenomenon of sensory biology. This effort is highly relevant also from a clinical point of view. Exposure to loud sounds is the leading cause of hearing impairment and also affects synaptic sound encoding (hidden and overt hearing loss; Kujawa & Liberman, 2015; Moser & Starr, 2016). Hearing impairment reduces the range of audible sound pressures likely by various mechanisms. Hearing aids and cochlear implants, key means for partially restoring auditory function, face the problem of mapping the entire range of relevant sound pressures to the limited dynamic range of the diseased auditory system.

## Main: heterogeneity of afferent synapses

### Molecular and structural heterogeneity

To set the stage for reviewing candidate mechanisms potentially contributing to sound intensity encoding, we start by reviewing the puzzling phenomenon that the small and compact IHCs harbor afferent synapses that vary dramatically in structure and function. The AZ of these synapses anchors the so‐called synaptic ribbon or dense body, a proteinaceous structure that tethers a halo of synaptic vesicles near the active zone (see model in Fig 1 and schematic in Fig 2C; Matthews & Fuchs, 2010; Lagnado & Schmitz, 2015; Moser *et al*, 2019). Structural differences among the afferent IHC‐SGN synapses at one tonotopic place or even within one IHC have already been noted by serial section electron microscopy studies that reported different ribbon size and shapes, synaptic membrane contacts, and postsynaptic fiber morphology (Spoendlin, 1969; Dunn, 1975; Liberman, 1980). Combining functional characterization of individual SGNs and backtracing their peripheral neurite to the afferent synapse, Charles Liberman related the physiological properties of SGNs to the position of their synaptic contacts with IHCs (Liberman, 1982). Merchan‐Perez & Liberman (1996) and later Kantardzhieva *et al* (2013) specifically compared the ultrastructure of synapses on the modiolar and the pillar side of IHCs. They found larger pools of membrane‐proximal and ribbon‐associated synaptic vesicles (SVs) at modiolar AZs that could also contain more than one ribbon, while the trend toward larger size of modiolar ribbons did not reach statistical significance in either study.

More recent volume electron microscopy studies used focused ion beam scanning electron microscopy (FIB‐SEM; Michanski *et al*, 2019) and surface block‐face scanning electron microscopy (SBEM; Hua *et al*, 2021) to reconstruct individual IHCs or ensembles of up to 20 IHCs, as well as the afferent and efferent connectivity in the organ of Corti of the mouse cochlea. Building on sample sizes of more than one hundred modiolar and pillar AZs, each, SBEM (Hua *et al*, 2021) demonstrated that pillar and modiolar ribbon volumes differed significantly and consistently across three mouse cochleae. Ribbons on the modiolar side were ~ 30% larger on average than the pillar ones: modiolar–pillar gradient of ribbon volume (Fig 4). Both studies demonstrated the occurrence of multiple presynaptic ribbons at individual modiolar AZs. SBEM analysis also provided evidence for a conservation of overall ribbon material: the more ribbons a given IHC contained, the smaller were the ribbons on average. FIB‐SEM analysis of few IHCs also showed trends toward larger individual ribbons on the modiolar side, as well as greater total ribbon volume and more SVs per AZ (Michanski *et al*, 2019).

Some distributions of synaptic properties can also be examined using immunofluorescence studies, which offer ease of orientation, high throughput, large sample sizes, and identification of specific labeled proteins, yet at the expense of more limited spatial resolution. Juxtaposed immunofluorescence of pre‐ and postsynaptic markers allows efficient and safe identification of IHC ribbon synapses (Khimich *et al*, 2005) and has been used extensively in the field for counting and localizing them within IHCs and the organ of Corti of various species under physiological and pathological conditions (reviewed in Meyer & Moser, 2010; Rutherford, 2015; Wichmann & Moser, 2015). While most synaptic structures, such as SVs, ribbons, presynaptic density, and Ca^2+^ channel clusters, are below the resolution limit of confocal microscopy, superresolution techniques such as 4Pi (Hell & Stelzer, 1992), Stimulated Emission Depletion (STED) (Hell & Wichmann, 1994), and Minimal Photon Fluxes (MINFLUX) optical nanoscopy (Balzarotti *et al*, 2017) enable a more detailed quantification of synaptic molecular nanoanatomy. For semi‐quantitative assessment of the abundance of synaptic proteins, analyses of immunofluorescence intensity, 3D‐integrated intensity, area, and volume of immunofluorescent spots have been employed for confocal imaging. The caveat of investigated structures being below the resolution limit needs to be considered when interpreting estimates of diameter, area, or volume obtained by confocal imaging of immunofluorescence. Additionally, we need to consider that studies can differ in their definition of the pillar and modiolar halves of the basolateral IHC pole (e.g., Meyer *et al*, 2009; Liberman *et al*, 2011).

**Figure 5:**
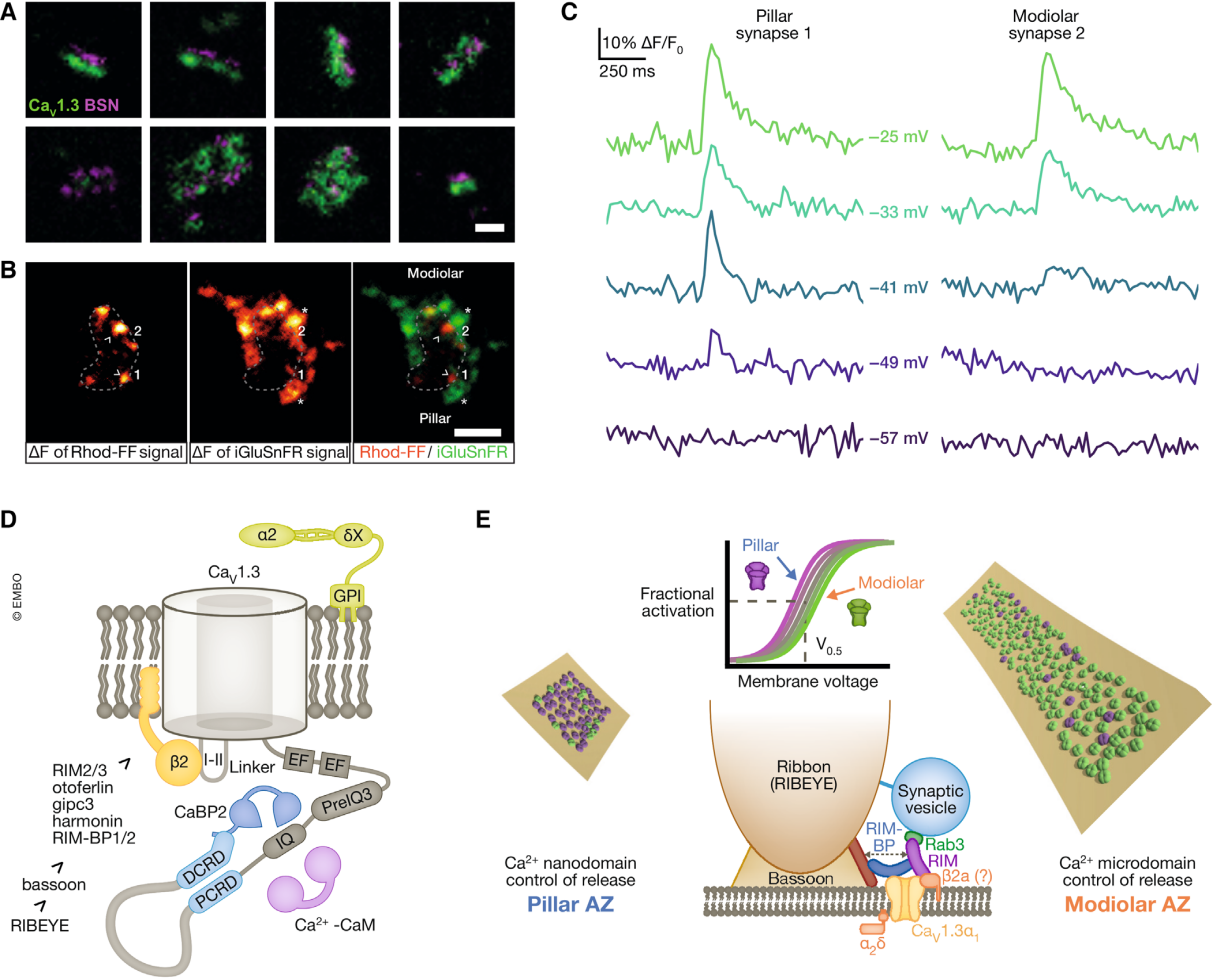
Position dependence of molecular structure and function of afferent synapses (A) STED nanoscopy reveals heterogeneous Ca_V_1.3 and bassoon clusters (modified from Neef *et al*, 2018). Scale bar: 200 nm. (B) Two‐color spinning disc confocal imaging of presynaptic Ca^2+^ signals (Rhod‐FF, arrowheads) and glutamate release (iGluSnFR in SGN membrane, asterisks. From Özçete & Moser, 2021). Scale bar: 5 μm. (C) Example traces of iGluSnFR imaging showing release dynamics of two synapses shown in B labeled “1” and “2” innervating the same IHC from either pillar or modiolar side. Note that pillar synapse 1 is already active at lower voltages than modiolar synapse 2 (from data used in Özçete & Moser, 2021). (D) Ca_V_1.3 (α1_D_ or Ca_V_1.3α1 pore‐forming subunit) constitutes ≥ 90% of all IHC Ca^2+^ channels and is present in splice variants. They differ in the length of the cytosolic C‐terminus that contains several domains for autoregulation and modulation by interacting proteins listed in the panel. (E) IHC AZs differ in the number and functional properties of their Ca^2+^ channels as well as in the coupling of Ca^2+^ influx to SV exocytosis: pillar AZs (left) contain fewer Ca^2+^ channels that activate at more negative potentials (“magenta” channels, see top, middle panel) and exert a so‐called Ca^2+^ nanodomain control of exocytosis (tighter coupling, see bottom, middle panel). Modiolar AZs (right) are more heterogeneous, but on average contain more Ca^2+^ channels that activate at less negative potentials (“green” channels) and exert a so‐called Ca^2+^ microdomain control of exocytosis (looser coupling). Illustration based on material from Pangrsic *et al* (2018) and Özçete & Moser (2021), not drawn to scale.

Nonetheless, confocal immunofluorescence studies have provided converging evidence for a modiolar–pillar gradient of decreasing ribbon size and Ca^2+^ channel number of IHC AZs in mice (Meyer *et al*, 2009; Liberman *et al*, 2011; Ohn *et al*, 2016; Figs 4 and 5), which, for ribbon size, agrees with the electron microscopy observations (Merchan‐Perez & Liberman, 1996; Kantardzhieva *et al*, 2013; Michanski *et al*, 2019; Hua *et al*, 2021). Moreover, the gradient of the AZ Ca^2+^ channel complement derived from immunofluorescence could be corroborated by *ex vivo* functional imaging of Ca_V_1.3‐driven Ca^2+^ signals at single AZs in separate experiments (Ohn *et al*, 2016; Figs 4 and 5). The maximal amplitude of Ca_V_1.3‐driven Ca^2+^ signals also positively correlated with ribbon size estimates in the same confocal recording (Frank *et al*, 2009). This was based on labeling of ribbons with a ribbon‐binding fluorescent peptide, using its fluorescence intensity to approximate ribbon size (Frank *et al*, 2009) which likewise decreased along a modiolar–pillar gradient (Ohn *et al*, 2016). Confocal and superresolution immunofluorescence imaging in mouse IHCs also served the further dissection of the molecular nanoanatomy and physiology of IHC AZs. Examples include the Ca_V_1.3 Ca^2+^ channels (Frank *et al*, 2010; Wong *et al*, 2014; Neef *et al*, 2018; Fig 5A), as well as the multidomain AZ proteins bassoon (Fig 5A; Khimich *et al*, 2005; Frank *et al*, 2010; Jing *et al*, 2013; Neef *et al*, 2018), piccolo/piccolino (Khimich *et al*, 2005; Müller *et al*, 2019; Michanski *et al*, 2023), rab‐binding molecule (RIM) (Jung *et al*, 2015; Picher *et al*, 2017b), and RIM‐binding protein (RBP; Krinner *et al*, 2017, 2021). This has led to the concept that clusters of Ca_V_1.3, bassoon, RIM, and RBP are mostly assembled in the shape of stripes, next to less prevalent double stripes, spot‐like, and more complex protein assemblies (Fig 5A). A comprehensive account on the abundance and topography of AZ proteins as a function of synapse position has yet to be established and should then be related to synapse function.

### Functional heterogeneity

**Figure 6:**
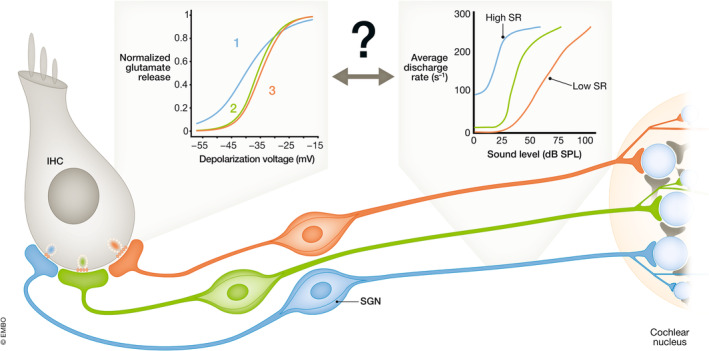
How do afferent synapse subtypes and SGN subtypes relate to each other? Color code and insets tentatively relate putative ribbon synapse subtypes and the SGN subtypes: this hypothesis needs further experimental and theoretical investigation. Illustration based on material from Özçete & Moser (2021).

Major functional AZ heterogeneity was discovered even within individual IHCs across tonotopic positions (Frank *et al*, 2009; Meyer *et al*, 2009). Specifically, when studying presynaptic Ca^2+^ signaling at single AZs, which is almost entirely mediated by Ca_V_1.3 channels (Platzer *et al*, 2000; Brandt *et al*, 2003; Fig 5D), striking differences in the voltage‐dependence of activation and maximal amplitude of Ca^2+^ signals were observed (Frank *et al*, 2009; Meyer *et al*, 2009; Fig 5E). Ca^2+^ imaging with low affinity Ca^2+^ indicators and added exogenous Ca^2+^ chelators approximates Ca^2+^ influx at individual AZs, but is limited in its temporal resolution (Frank *et al*, 2009). A quantification of nanophysiology and nanoanatomy of presynaptic Ca^2+^ channels found that the number of Ca^2+^ channels per AZ varied between 30 and 300 channels, organized in variably shaped clusters (Neef *et al*, 2018; Fig 5A). The average number of Ca^2+^ channels (mean of 125 and a median of 118: Neef *et al*, 2018) agrees well with previous estimates obtained from the whole‐cell Ca^2+^ current in hair cells (Roberts *et al*, 1990; Brandt *et al*, 2005). Some AZs showed multiple, or large morphologically complex, Ca^2+^ channel clusters, which are likely to represent AZs with many Ca^2+^ channels (Neef *et al*, 2018). While not demonstrated in these experiments due to limited optical resolution, they might represent AZs occupied by multiple ribbons (up to 3 in mature IHCs) that have been observed by electron microscopy and superresolution STED microscopy (Kantardzhieva *et al*, 2013; Wong *et al*, 2014; Michanski *et al*, 2019; Hua *et al*, 2021). Interestingly, most IHCs examined in *ex* vivo Ca^2+^ imaging experiments contained one AZ whose Ca^2+^ influx was substantially stronger than that of the others (“winner AZ” with 2.5 times greater amplitude than the average of the others; Ohn *et al*, 2016). Whether these represent multi‐ribbon AZs remains to be tested, e.g., by combining STED imaging of Ca^2+^ signals and ribbons. Functionally, large AZs with many Ca^2+^ channels and corresponding strong maximal Ca^2+^ signal tended to localize to the modiolar side (Figs 2, 3, 4, 5, 6). This modiolar–pillar gradient of decreasing AZ size and maximal Ca^2+^ influx (Ohn *et al*, 2016; Michanski *et al*, 2019; Hua *et al*, 2021) is seemingly at odds with the pillar–modiolar gradient of decreasing SR and acoustic sensitivity of SGNs (Liberman, 1982). In other words, provided comparable open probability and release site coupling of the channels, AZs with a larger number of Ca^2+^ channels should provide more synaptic release for a given IHC potential and would be expected to drive greater spontaneous rates of SGN firing (Wong *et al*, 2013).

A potential solution to this apparent conundrum came from studying the voltage‐dependent activation of the presynaptic Ca^2+^ influx. Here, it was found that AZ Ca^2+^ signals at the pillar side activate at lower voltages (i.e., hyperpolarized voltage) than the modiolar ones, resulting in a pillar–modiolar gradient of voltage‐dependent AZ activation by resting and receptor potentials of IHCs (Ohn *et al*, 2016; Figs 4, 5 and 6). This was quantified for the voltage of half‐maximal activation (V_0.5_) of the Ca^2+^ signals and reproduced in several studies since (Jean *et al*, 2019; Sherrill *et al*, 2019; Özçete & Moser, 2021; Cantu‐Guerra *et al*, 2023), leading to the hypothesis that the high SR and low sound threshold of SGNs synapsing on the pillar side are rooted in Ca^2+^ channel activation at the pillar AZs at resting and low receptor potentials (Ohn *et al*, 2016; Moser *et al*, 2019): Pillar AZs drive high SR SGNs, as their Ca^2+^ channels would be active already at the resting IHC potential (assumed to be approximately −55 mV) and be readily further recruited with small receptor potentials. In contrast, modiolar AZs which, consistent with the notion of them being presynaptic to low SR SGNs, would show no or very little activity at rest and need stronger receptor potentials to activate. As a next step to test this hypothesis, imaging of glutamate release from IHC AZs by virally expressing the genetically encoded glutamate sensor iGluSnFR in SGNs was employed (Fig 5B and C). Indeed, not only Ca^2+^ influx, but also the ensuing glutamate release from pillar AZ occurred at lower voltages (Fig 5C; Özçete & Moser, 2021). Yet, iGluSnFR imaging could not reliably detect release of individual SVs such that a distinction of high and low spontaneous rate of transmission synapses was not possible. However, recordings of excitatory postsynaptic currents (EPSCs) from modiolar and pillar synapses indicated higher rates of spontaneous release for pillar synapses which supports the above hypothesis (Siebald *et al*, 2023; preprint: Jaime Tobón & Moser, 2022).

The dual‐color imaging of Ca^2+^ signals and glutamate release of the same AZ allowed to obtain a first estimate of the apparent Ca^2+^ dependence of release on the single synapse level (Özçete & Moser, 2021). Previous work based on whole‐cell measurements of IHC Ca^2+^ influx and exocytic membrane capacitance increments (Brandt *et al*, 2005; Wong *et al*, 2014) in mouse IHCs after hearing onset had shown that the relationship between the two (apparent Ca^2+^ dependence of exocytosis) across all AZs followed a power function with a lower power (1.4) when probed by changing the number of contributing Ca^2+^ channels than the intrinsic Ca^2+^ dependence of exocytosis established with Ca^2+^ uncaging (4–5 ions binding to the Ca^2+^ sensor of SV fusion; Beutner *et al*, 2001) or the apparent Ca^2+^ dependence of exocytosis upon changes in single Ca^2+^ channel current (~ 3–4) (Brandt *et al*, 2005; Wong *et al*, 2014). This indicated that “nanodomain Ca^2+^”, contributed by few Ca^2+^ channels in immediate proximity of the vesicular release site, governs release (“Ca^2+^ nanodomain‐like control of exocytosis”). Experiments testing the effects of intracellular application of Ca^2+^ chelators with different binding rates (Moser & Beutner, 2000; Goutman & Glowatzki, 2007; Pangršič *et al*, 2015) further corroborate this hypothesis.

Moreover, paired pre‐ and postsynaptic patch‐clamp recordings, which allow recording of glutamate release from single IHC synapses, also indicated a Ca^2+^ nanodomain‐like control of exocytosis (Goutman & Glowatzki (2007) before hearing onset, but see Wong *et al* (2014) for a more Ca^2+^ microdomain‐like control of exocytosis at this developmental stage). However, since these recordings could only target single synapses, it had remained unclear whether this homogeneously applies to all AZs (Heil & Neubauer, 2010). This could now be tested by dual‐color imaging of Ca^2+^ signals and glutamate release that allows recording of the activity of multiple AZs in one IHC at different levels of depolarization. This revealed differences of the apparent Ca^2+^ dependence of release among the AZs from near linear (Ca^2+^ nanodomain‐like control) to supralinear (Ca^2+^ microdomain‐like control, where the combined activity of several channels in > 100 nm distance from the release site shape the Ca^2+^ signal that drives release). These findings suggest additional diversity of AZ structure and function beyond that of different number and gating of Ca^2+^ channels, i.e., differences in the spatial coupling of Ca^2+^ channels and release sites among AZs. Indeed, even if the Ca^2+^ channel density at the AZ was constant, a Ca^2+^ microdomain‐like control would be predicted for AZs with many Ca^2+^ channels due to overlap of the Ca^2+^ domains generated by the individual channels (Wong *et al*, 2014). Future optical nanoscopy (Grabner *et al*, 2022), electron microscopy (Wong *et al*, 2014; Chen *et al*, 2015; Nakamura *et al*, 2015; Chakrabarti *et al*, 2018, 2022; Butola *et al*, 2021), and computational modeling (Chapochnikov *et al*, 2014; Wong *et al*, 2014) work will be required to evaluate the scope of Ca^2+^ channel and release site topographies adopted by the diverse IHC AZs. This ideally will relate morphological and functional data on the AZ, e.g., from paired patch‐clamp and/or optical recordings, to synapse position within the IHC. Moreover, analysis of the initial rate of glutamate release prior to SV pool depletion will be important for faithfully assessing the Ca^2+^ dependence of release. This was not amenable to the dual‐color imaging of Ca^2+^ signals and glutamate release (Özçete & Moser, 2021), which limits the reliability of the approach and calls for validation by more resolved recordings. Future studies should also capture other key synaptic parameters such as SV tethering and docking, ideally at different functional stages (Chakrabarti *et al*, 2018, 2022), for which differences among AZs have not yet been studied in detail. Most importantly, the field will need to work on relating synaptic heterogeneity and neural firing diversity, which is far from trivial given the methodological differences of e.g. fluorescence imaging of glutamate release *ex vivo* and extracellular recordings from single SGNs *in vivo*.

A first attempt of relating functional properties of IHC AZs and SGN properties was recently done based on the above‐mentioned dual‐color imaging of Ca^2+^ signals and glutamate release of the same AZ (Özçete & Moser, 2021). Unbiased clustering of functional single synapse parameters indicated three synapse types (Fig 6). Cluster 1, with the most hyperpolarized operating range, near‐linear apparent Ca^2+^ dependence of release (indicating a tight Ca^2+^ nanodomain‐like coupling of Ca^2+^ influx and release sites of synaptic vesicles, SVs), and broadest dynamic range (change of potential from 10 to 90% of release), included all synapses of the pillar side of the IHCs (Fig 6).

Modiolar synapses contributed to all three clusters, whereby clusters 2 and 3 were similar in operating over a more depolarized, smaller range of potentials. They showed a supralinear apparent Ca^2+^ dependence with greater power for cluster 3, suggesting a looser Ca^2+^ microdomain‐like coupling of Ca^2+^ influx and SV release sites. It is tempting to speculate that synapses of cluster 1 drive high SR SGNs and that the other clusters might correspond to low and potentially intermediate SR SGNs. Yet, the dynamic range of the transfer function of cluster 1 synapses is the widest, while *in vivo* recordings show that the dynamic ranges of firing‐rate/sound‐level functions of high SR, low‐threshold SGNs are the narrowest. Possible explanations include the notion of partial SV pool depletion at pillar AZs at the more depolarized resting IHC potentials *in vivo* as well as postsynaptic saturation. Clearly, this framework remains to be tested by computational modeling and experiments that involve recordings of SGN activity such as the combination of pre‐ and postsynaptic patch‐clamp recordings or simultaneous imaging of SGN membrane potential. Recordings of EPSCs, in addition, provide access to the individual release events (Glowatzki & Fuchs, 2002) that are subject to regulation beyond Ca^2+^ triggering of SV fusion and, hence, offer another level of complexity (Grant *et al*, 2010; Rutherford *et al*, 2012; Chapochnikov *et al*, 2014; Huang & Moser, 2018; Niwa *et al*, 2021). Indeed, EPSCs of the afferent IHC‐SGN synapse vary greatly in amplitude and shape. While, regardless of this EPSC variability, > 95% of EPSCs successfully drive spike generation in the peripheral SGN neurite, shorten the spike latency and provide closer coupling of the spike to the time of the release (Rutherford *et al*, 2012). The EPSC variability has initially been attributed to more or less coordinated release of several SVs (coordinated multivesicular release; Glowatzki & Fuchs, 2002; Goutman & Glowatzki, 2007; Grant *et al*, 2010)). Alternatively, univesicular release through a dynamic fusion pore has been proposed to underly the different EPSC amplitudes and shapes (Chapochnikov *et al*, 2014; Grabner & Moser, 2018; Huang & Moser, 2018). Regardless of the precise underlying mechanism, the specifics of release likely co‐determine the SGN firing properties and are thus critical for sound encoding.

## Outlook—Synaptic heterogeneity

Relating molecular composition, structure, and function of afferent synapses and studying their position dependence remains an important task. Confocal and superresolution optical imaging of pre‐ and postsynaptic molecular nanoanatomy during physiological analysis or after immunofluorescent labeling will likely contribute here, as will electron microscopy and tomography. It is conceivable that the position‐dependent regulation of AZ size, Ca^2+^ channel number, and molecular topography will involve the abundance of scaffold proteins of IHC AZs such as bassoon (Frank *et al*, 2010), RIBEYE (Jean *et al*, 2018), RIM2 (Jung *et al*, 2015; Picher *et al*, 2017b), and RIM‐BP2 (Krinner *et al*, 2017, 2021) that undergo direct (RIM, RIM‐BP2) or indirect (bassoon, likely RIBEYE) interaction with Ca_V_1.3 Ca^2+^ channels (Fig 5D and E). Indeed, an alteration of Ca^2+^ channel clustering at IHC AZs was found for a deletion of any of them (Frank *et al*, 2010; Jung *et al*, 2015; Krinner *et al*, 2017; Jean *et al*, 2018). Interestingly, bassoon (Frank *et al*, 2010), but not RIM2a (Jung *et al*, 2015) or RIM‐BP2 (Krinner *et al*, 2017), seems required for establishing the normal variance of maximal synaptic Ca^2+^ influx of IHC AZs. Differences in the Ca^2+^ channel complexes (Fig 5D) between IHC AZs might originate from their subunit composition as well as alternative splicing of Ca_V_1.3α1 (Shen *et al*, 2006; Scharinger *et al*, 2015; Ohn *et al*, 2016; Vincent *et al*, 2017), which may influence binding of EF‐hand Ca^2+^ binding proteins (CaBPs, calmodulin; Grant & Fuchs, 2008; Schrauwen *et al*, 2012; Picher *et al*, 2017a; Oestreicher *et al*, 2021), multidomain proteins of the AZ (e.g., RIM‐BPs and RIM), and adapters (e.g., Gipc3, unpublished). For example, a genetic manipulation of the differentially spliced Ca_V_1.3α1 C‐terminus that is expected to abolish the long Ca_V_1.3α1 splice variant (Scharinger *et al*, 2015) indeed resulted in a mild alteration of Ca^2+^ influx at IHCs, but the functional relevance for sound encoding remains to be clarified (Ohn *et al*, 2016). Moreover, while the Ca_V_β2 subunit seems to prevail (Neef *et al*, 2009), other subunits are expressed, too (Kuhn *et al*, 2009; Neef *et al*, 2009). Interestingly, disruption of the Ca_V_α2δ2 subunit led to reduced Ca^2+^ influx and a loss of precise juxtaposition of pre‐ and postsynaptic structures at the afferent IHC synapse (Fell *et al*, 2016).

Resolving the topography of these molecular players and their complexes will benefit from advanced optical nanoscopy such as MINFLUX, which recently revealed such a molecular AZ nano‐map for rod photoreceptors (Grabner *et al*, 2022), or ONE expansion microscopy (preprint: Shaib *et al*, 2022). Freeze fracture immunolabeling of Ca^2+^ channels and AZ proteins followed by electron microscopy (Chen *et al*, 2015; Nakamura *et al*, 2015; Butola *et al*, 2021) offers an alternative/additional approach. Electron tomography allows the definition of SV subpools based on their tethering and docking status (Fernández‐Busnadiego *et al*, 2010; Chakrabarti *et al*, 2018). Comparative electron tomography of pillar and modiolar synapses, ideally following optogenetic stimulation and high‐pressure freezing (Chakrabarti *et al*, 2022), is expected to majorly advance our understanding of the nanoanatomy and nanophysiology of the IHC synapse. Since the postsynaptic density is typically well accessible in these samples, the presynaptic parameters and the size of the postsynaptic density (PSD) can be simultaneously approached, but typically not fully captured given the limit of typical 200 kV microscopes to a sample thickness of 250 nm or less. Another remaining challenge is to simultaneously analyze the topography of Ca^2+^ channels and SVs tethered or docked to the AZ membrane. Approaches such as 2‐color MINFLUX and cryo‐electron tomography promise progress toward this end.

Furthermore, efforts are required also to elucidate the molecular and structural knowledge regarding the PSD of the postsynaptic SGNs. Immuno‐electron microscopy and confocal and superresolution immunofluorescence have established the notion that a ~ 0.5–1.5 μm sized dense array of glutamate receptors with a ring‐like structure likely surrounding the presynaptic AZ (Matsubara *et al*, 1996; Meyer *et al*, 2009; Wong *et al*, 2014; Rutherford *et al*, 2023) ensures efficient detection of glutamate. A number of postsynaptic density proteins such as PSD‐95, Shank, and Homer1 have been identified (e.g., Davies *et al*, 2001; Reijntjes *et al*, 2020). Few studies have addressed the question whether postsynaptic elements show gradients along the pillar–modiolar axis. Interestingly, opposing size gradients (decreasing in pillar–modiolar direction for AMPA receptor clusters, but in modiolar–pillar direction for ribbon size) have been described for IHCs of CBA mice (Liberman *et al*, 2011; Reijntjes *et al*, 2020), but while the presynaptic gradient was consistently found in two other mouse lines, the postsynaptic gradient was not (Reijntjes *et al*, 2020). A recent detailed study of the PSD of the IHC‐SGN synapse in C57BL/6J mice revealed a modiolar–pillar gradient of PSD size and showed that the GluA3 is required for modiolar–pillar gradient of ribbon size (Rutherford *et al*, 2023).

Together, pre‐ and postsynaptic molecular and ultrastructural properties co‐determine sound encoding at a given IHC ribbon synapse. However, established neuronal concepts such as synaptic strength might fall short when it comes to describing the heterogeneous synapses of IHCs that are driven by graded receptor potentials, rather than action potentials. Instead, characterizing threshold as well as resting and maximal rates of synaptic transmission as a function of IHC potential in *ex vivo*, but ideally also *in vivo* experiments, will serve our understanding of sound intensity coding better. The parameter of maximal presynaptic strength, i.e., the maximal glutamate release for strong depolarizations, likely will apply only to *ex vivo* analysis enabling such IHC stimulation.

Last not least, we propose to employ computational modeling to reconcile the various data on synaptic properties and to study how they relate to sound intensity coding by SGNs. Preliminary modeling results (Gabrielaitis, 2015) indicate that the voltage‐dependent activation of Ca_V_1.3 Ca^2+^ channels can explain much of the diversity of the spontaneous and sound‐evoked SGN firing (Peterson & Heil, 2021). For example, the difference in the threshold for Ca^2+^ channels—some above the IHC resting potential resulting in little or no Ca^2+^ triggered glutamate release and others at > 10% of activation at rest with substantial release—can readily account for the broad SR range of SGNs (~ 2 orders of magnitude). Modeling will also help evaluating the impact that Ca^2+^ channel‐release site coupling has on sound encoding. Different from the effects of voltage dependence of synapse function, this seems less intuitive. Generally, one could assume that large AZs with many Ca^2+^ channels that operate in a more depolarized voltage range are able to grade the number of open channels (product of total number of channels and open probability) and hence release over a wider voltage range. However, this notion implies a Ca^2+^ nanodomain‐like control of release (i.e., a linear apparent Ca^2+^ dependence of release), which might not be the case for these large synapses, where domain overlap among the many Ca^2+^ channels is likely to occur. A supralinear apparent Ca^2+^ dependence could compress the synaptic transfer function as is actually observed in clusters 2 and 3 of the IHC AZs. Yet, this seems to conflict with the notion of these synapses driving low SR SGNs that generally code a wider dynamic range of sound intensity. So, modeling will be critical to better understand the complex interplay of synaptic properties, excitability, and spike generation of type I SGN and to evaluate the relative contributions of these processes to diversify SGN sound intensity coding. In fact, studies have indicated that differences in SGN excitability exist and might be related to the molecular profiles type Ia–c (Crozier & Davis, 2014; Smith *et al*, 2015; Markowitz & Kalluri, 2020).

## Candidate signaling mechanisms for generating heterogeneous afferent synapses

**Figure 7:**
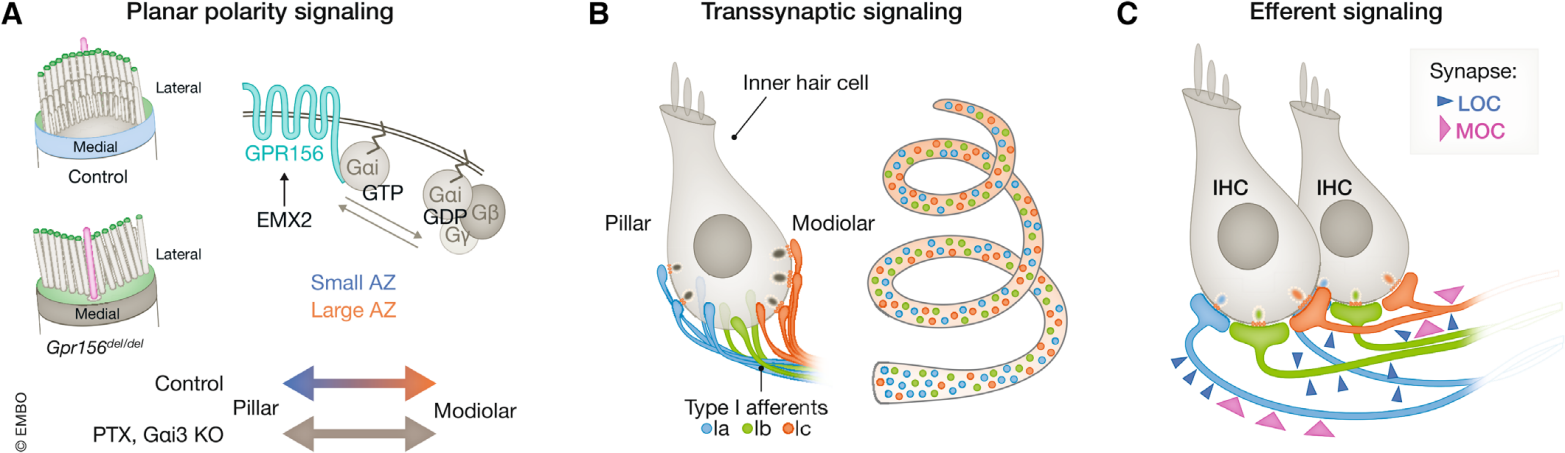
Candidate signaling mechanisms for establishing heterogeneous properties of IHC active zones (A) Establishment of apical planar polarity (top left) depends on Gai signaling triggered by GPR156 (top right). IHC expression of PTX and KO of Gai3 collapses the basolateral modiolar–pillar gradient of AZ size (modified from Kindt *et al*, 2021). (B) RNAseq data of several laboratories have revealed three type I SGN subtypes that have been proposed to correspond to high SR (Ia), intermediate SR (Ib), and low SR (Ic) SGNs. Those types occur all along the tonotopic axis (right). The transcriptional profile likely relates to the molecular SGN physiology and could also differentially instruct the properties of presynaptic AZs in IHCs via transsynaptic signaling. Indeed, postnatal disruption of the transcription factor Pou4f1, expressed in Ic (and less in Ib) collapsed the modiolar–pillar gradient of maximal AZ Ca^2+^ signaling (Modified from Shrestha *et al*, 2018). (C) The source (LOC vs. MOC) and extent of efferent innervation differs between SGNs and appears to balance the maximal afferent synaptic strength. Lesion of the efferent projection led to a collapse of the modiolar–pillar gradient of AZ size (Modified from Hua *et al*, 2021).

How could a small and compact sensory cell like an IHC establish such synaptic diversity? At least 4 candidate signaling mechanisms come to mind: (i) cell‐ or tissue‐level planar polarity, known to establish proper hair bundle orientation; (ii) transsynaptic signaling by molecularly diverse type I SGNs; (iii) direct or indirect signaling by efferent olivocochlear neurons; and (iv) position‐dependent impact of supporting cells. Figure 7 summarizes existing evidence for a contribution of the first three mechanisms.

### Cell‐ or tissue‐level planar polarity

Correct orientation of stereocilia bundles, which are deflected by the mechanical stimuli for gating of mechanotransducer ion channels, perpendicular to the pillar–modiolar axis is essential for efficient IHC activation by sound. This raises the possibility that planar polarity signaling, involved in setting proper hair bundle orientation at the apical hair cell membrane, might also contribute to determine synaptic properties at the base of the IHC along the pillar–modiolar axis. Indeed, interference with intrinsic (cell‐autonomous) planar polarity signaling by misexpression of pertussis toxin disrupts both proper hair bundle orientation (Tarchini *et al*, 2013) and the modiolar–pillar gradients of AZ size and maximal Ca^2+^ influx (Jean *et al*, 2019). It will be interesting to address the roles of GPR156, a recently identified orphan G‐protein coupled receptor (Kindt *et al*, 2021), as well as those of Gai1 and Gai3 that operate downstream of GPR156 (Kindt *et al*, 2021) in this regard. In addition, it will be interesting to analyze the effects on synapse organization of altering core‐planar cell polarity by disrupting Vangl1 and Vangl2 (Stoller *et al*, 2018).

### Transsynaptic signaling by molecularly diverse type I SGNs

Deletion of the transcription factor Pou4f1, which is expressed in type Ic (and less of Ib) SGNs that preferentially insert on the modiolar IHC side (Shrestha *et al*, 2018; Sherrill *et al*, 2019), collapsed the modiolar–pillar gradient of the maximal synaptic Ca^2+^ influx (Sherrill *et al*, 2019). This suggests that SGNs with a transcriptional program controlled by Pou4f1 can instruct properties of modiolar AZs. We note that the gradient of voltage‐dependent activation of Ca^2+^ channels was not affected, but, peculiarly and for unknown reasons, the ribbon size gradient was inverted (pillar–modiolar) in both control and *Pou4f1*‐deficient mice (Sherrill *et al*, 2019). It will now be interesting to also target other transcription factors, such as Runx1, which are thought to co‐determine the Ib and Ic molecular profile. Interestingly, disruption of Runx1 led to a collapse of the modiolar–pillar ribbon size gradient, too (Shrestha *et al*, 2023). Combining disruption of Runx1 function or misexpression of *Runx1* across all type I SGNs with molecular tagging of synapses formed by type Ib and Ic neurons will enable *ex vivo* studies of the impact of Runx1 on the properties of afferent IHC synapses and SGN excitability. Parallel *in vivo* recordings of spontaneous and sound‐evoked SGN firing will allow to relate alterations in position‐dependent AZ properties to potential changes in functional SGN diversity. However, so far the molecular cues mediating the transsynaptic signaling are unknown. One attractive candidate to investigate here are Ca_V_α_2_δx Ca^2+^ channel subunits (Fig 5D): a misalignment of AZ and postsynaptic density was demonstrated for Ca_V_α_2_δ2‐deficient IHCs (Fell *et al*, 2016), but no position‐dependent analysis of synaptic features has been performed yet. The tetraspan protein clarin‐1, interacting with harmonin, has also been proposed as a possible member of the IHC transsynaptic adhesion complex (Dulon *et al*, 2018). While there is some preliminary work on the function of synaptic adhesion proteins, such as neurexins (Sons *et al*, 2006) and neuroligins (Hoon *et al*, 2009; Ramirez *et al*, 2022), much remains to be done to elucidate the molecular players for implementing synaptic heterogeneity.

### Efferent signaling

Efferent innervation has been shown to differentially modulate afferent synapses (Ruel *et al*, 2001; Yin *et al*, 2014; Hua *et al*, 2021). Aside from acetylcholine, the efferent terminals employ dopamine and peptidergic transmitters and the precise effects of efferent modulation remain to be studied (Fuchs & Lauer, 2019). Efferent innervation approaching the unmyelinated peripheral SGN neurites is more prevalent near the modiolar IHC side (Liberman *et al*, 1990), which likely reflects the greater number of afferent synapses found there as well as a greater density of efferent synapses. Reconstruction of an organ of Corti volume spanning ≥ 15 IHCs revealed that efferent innervation of SGNs varies with their afferent innervation and synaptic location (Hua *et al*, 2021). The number of efferent synapses ranged from 0 to 20 per SGN, lower for SGNs with input from an AZ with a single ribbon, higher for SGNs facing multi‐ribbon AZs (primarily modiolar), and highest for SGNs with branched neurites driven by multiple AZs. It is tempting to speculate that this reflects a balance of the afferent input from IHC AZs by efferent modulation. SGNs contacting modiolar AZs received efferent input primarily from lateral olivocochlear neurons, whereas medial olivocochlear neurons formed *en passant* synapses mostly onto SGNs contacting pillar AZs, which could offer an additional means of differential efferent modulation of SGNs. Surgical lesioning of olivocochlear projections at the floor of the 4^th^ ventricle collapsed the modiolar–pillar gradient of ribbon size (Yin *et al*, 2014). It will be interesting in future experiments to study how the loss of efferent innervation or the loss of efferent transmission affects the afferent synaptic organization and SGN firing properties and whether the effects of disrupting transmission from lateral or medial olivocochlear neuron differ.

## Relating afferent synaptic heterogeneity and function to spiral ganglion neuron diversity

Currently, the field lacks approaches that could connect the exciting research on the diversity of SGN physiology, their molecular profile, and the presynaptic input. Bridging gaps between the different approaches and relating the likely intertwining candidate mechanisms will be key to generating a unified concept on sound intensity coding in the cochlea. For example, the tempting hypothesis that IHCs use heterogeneous AZs to decompose sound intensity information into complementary neural codes represented by the functionally and molecularly diverse type I SGNs awaits experimental and theoretical testing. Detailed biophysical modeling of sound encoding at the IHC‐SGN synapse demonstrated that the variation of a single parameter, the voltage of half‐maximal activation of Ca_V_1.3 channels, suffices to explain most, if not all, of the experimentally observed diversity of spontaneous and sound‐evoked SGN firing, despite the use of a simple and unvaried implementation of spike generation (Gabrielaitis, 2015). While not addressing likely contributions of divergent SGN excitability, these modeling results emphasize the impact of the voltage dependence of Ca^2+^ channel activation and likely of Ca^2+^ channel‐release site coupling.

**Figure 8:**
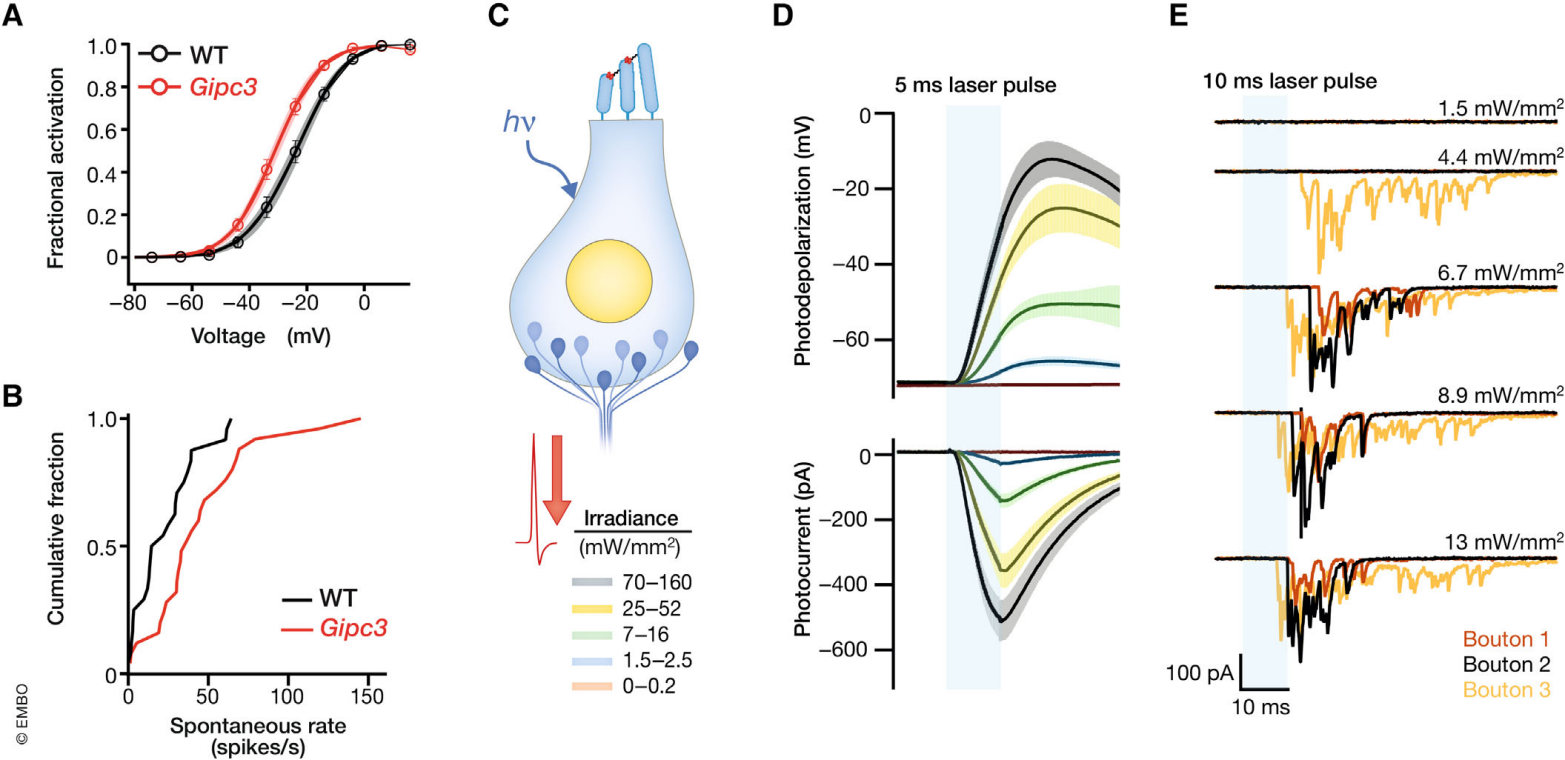
Relating afferent synaptic heterogeneity and spiral ganglion neuron diversity (A) Ca^2+^ influx in IHCs of *Gipc3* mutant mice shows a more negative activation range. (B) Increased spontaneous firing rate of *Gipc3* mutant mice (Modified from Ohn *et al*, 2016). (C–E) Optogenetic approach to mapping the synaptic position of functionally distinct SGNs on IHCs (C): ChR2‐H134R‐mediated IHC photocurrents lead to robust photodepolarization (D) and ensuing glutamate release revealed by recordings of light‐evoked EPSCs from postsynaptic boutons of SGNs (E). This way, IHCs can be stimulated in a less invasive way than using patch clamp (Adapted from Chakrabarti *et al*, 2022).

A first attempt to bridge *ex vivo* synaptic physiology and *in vivo* sound encoding took advantage of a mouse mutant with loss of function for the adapter protein Gipc3 (GAIP interacting protein, C‐terminus 3), a PDZ protein and candidate regulator of Ca^2+^ channels. Defects of the human *GIPC3* gene cause deafness (Charizopoulou *et al*, 2011; Rehman *et al*, 2011) and *Gipc3* disruption in mice led to audiogenic seizures and progressive hearing loss (Charizopoulou *et al*, 2011). Early‐onset hearing impairment in *Gipc3* mutant mice has been attributed to dysfunction of hair cell stereocilia, although its localization within hair cells resembles a cytoplasmic pattern similar to myosin VI (Charizopoulou *et al*, 2011). In the brain, glutamate release was shown to depend on interaction between myosin VI and the Gipc3 homolog Gipc1 (Giese *et al*, 2012). Analysis of *Gipc3*‐deficient IHCs revealed increased Ca^2+^ influx and exocytosis (Ohn *et al*, 2016). Ca^2+^ channel activation showed on average a hyperpolarized shift of the voltage of half‐maximal activation (Fig 8A, −7 mV on average for all AZs) while maintaining the pillar–modiolar gradient of the voltage‐dependent activation of individual AZs.

Interestingly, a pillar–modiolar gradient was also observed for the maximal synaptic Ca^2+^ influx in Gipc3‐deficient IHCs, contrasting the modiolar–pillar gradient typically found in wild‐type IHCs (Fig 4). This resulted in the unique scenario that both mechanisms of synaptic strength coincided at pillar synapses in addition to the overall activation of Ca^2+^ influx at lower voltages. Should the presynaptic hypothesis of the functional SGN diversity be correct, one would expect higher spontaneous firing rates and lower sound thresholds of SGNs in *Gipc3* mutant mice. In keeping with this hypothesis, the mutants showed substantially elevated spontaneous firing rates (Fig 8B) as well as onset firing rates upon suprathreshold sound stimulation (Ohn *et al*, 2016). Interpretation of the greater spontaneous rate is challenging, given that (i) impaired mechanotransduction resulted in elevated sound thresholds hampering the analysis of sound encoding by SGNs and (ii) Ca^2+^ influx at pillar AZs of *Gipc3*‐deficient IHCs showed both activation at lower voltages and greater maximal amplitude. Interestingly, a Ca_V_1.3 (Cacna1d) mutation that was aimed to abolish the function of splice variants with long C‐terminus (Scharinger *et al*, 2015) generated greater Ca^2+^ influx amplitude across all IHC synapses (comparable to Gipc3 mutation) but, unexpectedly, did not alter its voltage‐dependent activation (Ohn *et al*, 2016). The normal opposing gradients of voltage‐dependent activation and maximal amplitude of synaptic Ca^2+^ influx were maintained. Interestingly, *in vivo* SGN recordings in these Ca_V_1.3 mutant mice revealed a normal distribution of spontaneous firing rates, normal sound‐evoked firing rates, and normal sound‐pressure dependence of firing. The comparison of the two mutants seems to indicate a greater impact of the voltage dependence of Ca^2+^ influx for determining SGN firing behavior.

## Outlook—relating synaptic heterogeneity and functional spiral ganglion neuron diversity

Considering the challenge to bridge the gap between *ex vivo* and *in vivo* analysis of sound intensity coding, we provide some more but not exhausting suggestions on future approaches:

### Harmonize protocols to characterize synaptic transmission *in* and *ex vivo* as closely as possible

*In vivo* recordings from single SGNs have routinely accommodated the stochastic nature of single active zone function with few vesicular release sites by applying multiple repetitions of a given stimulus. Yet, often they did not fully scrutinize synaptic functions such as presynaptic pool dynamics. *Ex vivo* recordings have often (i) lumped together all synapses of an IHC by whole‐cell recordings of Ca^2+^ and exocytic membrane capacitance changes, (ii) studied immature IHCs, (iii) used unphysiological temperature and extracellular Ca^2+^ concentration, (iv) lacked sensitivity or kinetics, and/or (v) presented stimuli with few repetitions if repeated at all, which reports stochastic realizations given the few vesicular release sites per AZ. In the future, electrophysiological or optical recordings of spontaneous synaptic transmission or spontaneous firing rate from individual synapses, ideally combined with identification of the molecular SGN profile, will enable to tentatively assign synaptic properties to type I SGN functional or molecular subpopulations. Moreover, the use of more intact *ex vivo* preparations (Jagger & Housley, 2002; Chan & Hudspeth, 2005; Jean *et al*, 2020) as well as physiological temperature and extracellular Ca^2+^ concentration will help bridging the gaps. Finally, taking advantage of approaches such as optogenetics will allow to probe synaptic transmission in a less invasive way (Fig 8C–E) in more intact preparations, in particular when combined with optical readout of SGN activity, which is still to be established.

### Analyze mouse mutants with altered synaptic properties and/or synaptic heterogeneity

Clearly, analyzing mouse mutants such as mice carrying gain of function mutations of Ca_V_1.3 (Pinggera *et al*, 2015), with activation at lower voltage and, hopefully, better preserved acoustic sensitivity than found in *Gipc3* mutants, will be helpful to test the hypothesis of presynaptic determination of SGN firing diversity. Ideally, aside from *ex* and *in vivo* physiology, SGN RNA sequencing should be considered to reveal potential changes in SGN molecular profile or the SGN subtype representations. Glutamatergic transmission at the afferent IHC‐SGN synapse ‐ employing the vesicular glutamate transporter Vglut3 ‐ is required for maintaining the molecular SGN subtype specification (Shresthra *et al*, 2018; Sun *et al*, 2018). Interestingly, disruption of glutamatergic IHC transmission by Vglut3 knock‐out or largely abolishing IHC exocytosis by mutation of otoferlin did not majorly alter the heterogeneity of presynaptic AZs (Karagulyan & Moser, 2023). This suggests that neither afferent synaptic activity nor proper subtype identity of SGNs are strictly required for establishing and maintaining presynaptic heterogeneity. However, we note that the Ca^2+^ influx activated at lower voltages in IHCs of both Vglut3 and otoferlin mutants and that the voltage‐dependence of Ca^2+^ influx was less variable in otoferlin‐mutant IHCs. Clearly more work, such as manipulating the SGN molecular profile by disruption or misexpression of key transcription factors will be required to study the consequences on afferent synaptic or neurophysiological SGN properties.

### Map the synaptic insertion of IHCs of low, intermediate, and high SR SGNs

The heroic serial section electron microscopy of cochleae tracing physiologically characterized SGNs back to their contact with IHCs (Liberman, 1982) has sculpted our view of IHC‐SGN connectivity and provided evidence for a synaptic origin of the functional SGN diversity (Merchan‐Perez & Liberman, 1996; Kantardzhieva *et al*, 2013). Surface block scanning electron microscopy (Hua *et al*, 2021) and light sheet fluorescence microscopy (Keppeler *et al*, 2021; Rankovic *et al*, 2021) now offer powerful new approaches to unravel the connectivity in the cochlea and, combined with labeling of single SGNs after *in vivo* recordings, might substantially expedite the process, promising larger numbers of characterized and backtraced SGNs.

### Computational modeling of sound encoding for reconciling data and evaluating impact of parameters

Clearly, modeling has the capacity to cover and bridge the different levels of observation and will likely be key to arrive at a unifying account of sound intensity coding. Yet, in order for it to scrutinize hypotheses, it requires reliable and detailed experimental results.

Together, the opportunities offered by new methods and concepts allow for work toward a better understanding of the principles of sound intensity coding. This would not only be a great step forward toward understanding how the auditory system works but would also be informative regarding human deafness and the effects of noise‐induced hearing loss. Furthermore, it would allow more targeted approaches for advanced hearing restoration, such as by optogenetic stimulation, where specific targeting of SGN subtypes could vastly improve the dynamic range that can be addressed by future optogenetic cochlear implants. Eventually, it will be exciting to relate such work on the auditory system to parallel studies of wide dynamic range intensity coding in other sensory systems. Vestibular function and vision, also employing ribbon synapses of secondary sensory cells, will be of particular interest.

## Author contributions

**Tobias Moser:** Conceptualization; supervision; funding acquisition; validation; investigation; visualization; writing – original draft; project administration; writing – review and editing. **Nare Karagulyan:** Formal analysis; investigation; methodology; writing – original draft; writing – review and editing. **Jakob Neef:** Formal analysis; supervision; visualization; writing – original draft; writing – review and editing. **Lina María Jaime Tobón:** Conceptualization; formal analysis; validation; investigation; visualization; methodology; writing – original draft; writing – review and editing.

In addition to the CRediT author contributions listed above, the contributions in detail are:

TM and LMJT prepared the first draft of the MS, all authors contributed to writing and editing, and JN and TM assembled the figures.
